# Comparative Analysis of Refractive Outcomes Following Cataract Surgery Using IOL Master 500 and IOL Master 700 Biometry Devices: A Retrospective Analysis

**DOI:** 10.3390/jcm13175125

**Published:** 2024-08-29

**Authors:** Sebastian Arens, Daniel Böhringer, Thabo Lapp, Thomas Reinhard, Sonja Heinzelmann-Mink

**Affiliations:** 1 Eye Center, Medical Center, Faculty of Medicine, University of Freiburg, 79106 Freiburg im Breisgau, Germany; daniel.boehringer@uniklinik-freiburg.de (D.B.); thabo.lapp@augen-franzsikus.de (T.L.); thomas.reinhard@uniklinik-freiburg.de (T.R.); sonja.heinzelmann@uniklinik-freiburg.de (S.H.-M.); 2 Department of Ophthalmology, St. Franziskus Hospital, 48145 Muenster, Germany

**Keywords:** cataract surgery, refractive outcomes, biometry, IOL Master 500, IOL Master 700, IOL calculation

## Abstract

**Background:** This study aims to compare the refractive outcomes of cataract surgery using two different biometry devices, the IOL Master 500 and IOL Master 700, and to investigate the influence of patient-related factors on these outcomes. **Methods:** In this retrospective study, we analyzed data from 2994 eyes that underwent cataract surgery. Multiple linear regression analyses were performed to examine the impact of the biometry device (IOL Master 500 or IOL Master 700), patient age, time elapsed between biometry and surgery, gender, and insurance status, as well as biometric parameters (anterior chamber depth, axial length, and corneal curvature), on postoperative refractive outcomes, specifically the deviation from target refraction. **Results:** The choice of the IOL Master device did not result in a statistically significant difference between the two devices (*p* = 0.205). Age (*p* = 0.006) and gender (*p* = 0.001) were identified as significant predictors of refractive outcomes, with older patients and males experiencing slightly more hyperopic outcomes compared to younger patients and females, respectively. The time elapsed between biometry and surgery and insurance status did not significantly influence the refractive outcomes. **Conclusions:** Our study, supported by a large cohort and a diverse group of patients representing typical anatomical variants seen in cataract surgery, supports the thesis that the IOL Master 500 and IOL Master 700 can be regarded as equivalent and effective for biometry in cataract surgery. The differences between the devices were negligible. Therefore, switching between the devices is safe for bilateral patients.

## 1. Introduction

Cataract surgery with intraocular lens (IOL) implantation is one of the most commonly performed surgical procedures worldwide to restore visual acuity in individuals with cataracts. Achieving precise refractive outcomes following cataract surgery is imperative to enhance patient satisfaction and minimize the need for postoperative visual aids like glasses or contact lenses. Accurate biometric measurements of the eye, encompassing axial length and corneal power, are pivotal for determining the appropriate IOL power to achieve the targeted postoperative refraction.

Two widely used biometry devices, the IOL Master 500 and the IOL Master 700, employ advanced optical technology to capture various biometric parameters, providing necessary information in order to accurately calculate IOL power. The IOL Master 500 utilizes partial coherence interferometry (PCI), while the IOL Master 700 uses swept-source optical coherence tomography (SS-OCT). SS-OCT offers several advantages over PCI, including superior depth resolution, faster acquisition speeds, and reduced susceptibility to signal attenuation in highly scattering tissues.

Previous studies have investigated the agreement between the IOL Master 500 and IOL Master 700 in terms of biometric measurements. Song et al. found high agreement between the devices, with no difference in predicting refractive outcomes after cataract surgery in a subgroup of 54 eyes [1]. Jiang et al. also reported good reliability between the two instruments for various biometric parameters in a study of 518 eyes [2]. However, these studies were limited by relatively small sample sizes due to a prospective approach and focused primarily on the alignment of biometric data rather than postoperative refractive outcomes.

While these studies provide valuable insights into the agreement between the IOL Master 500 and IOL Master 700, there is a lack of large-scale, real-world studies comparing the refractive outcomes of cataract surgery using these devices. Additionally, the influence of patient-related factors, such as age, gender, and insurance status, on refractive outcomes has not been extensively investigated in the context of these biometry devices.

Our hospital possesses both IOL Master 700 and IOL Master 500 devices, necessitating research to determine whether the measurements and refractive outcomes obtained from these devices are interchangeable. Establishing the interchangeability of these devices is crucial for ensuring consistent and reliable results, regardless of which device is used for a particular patient. 

This study aims to address these gaps by comparing the refractive outcomes of cataract surgery patients when employing the IOL Master 500 and IOL Master 700 for biometry in a large, real-world cohort. Furthermore, we investigate the influence of patient-related factors, including age, gender, time elapsed between biometry and surgery, and insurance status, on refractive outcomes. By utilizing a retrospective approach with clinical standard data, we introduce an unconventional method for determining the alignment of different devices for IOL power calculation in a large patient cohort, which has not been performed in previous studies.

The insights gained from this study will provide valuable information for optimizing cataract surgery outcomes and possibly selecting the most suitable biometry device for individual patients. Additionally, understanding the impact of patient-related factors on refractive outcomes will help surgeons tailor their approach to achieve the best possible results for each patient. With the knowledge of the range of the most likely postoperative refraction, it might be a further step for better individual patient guidance in IOL choice.

### Differences between the IOL Master 500/700

Before optical devices were established, ultrasound biometry was state-of-the-art for pre-surgery biometry. However, there are disadvantages regarding these measurements. In order to measure axial length, the ultrasound probe has to make contact with the cornea, which can affect the measurements and could potentially harm the corneal surface. Furthermore, the measurement is dependent on the skills of the performing healthcare provider. Also, depending on patient compliance, acquisition time is another factor to be considered. Although these factors have to be considered, ultrasound biometry is still a valid method for IOL calculation, as was found by Haigis et al. [3]. Meanwhile, another investigation by Connors et al. suggests that there is an advantage in reproducibility and accuracy using partial coherence interferometry instead of ultrasound [4]. Eleftheriadis was able to find similar results, suggesting an advantage for partial coherence tomography over ultrasound biometry in 100 cases [5]. The superiority of IOL Master device measurements, especially considering the experience of the performing physician, was investigated by Findl et al. [6].

The IOL Master 500 and 700 are advanced ophthalmic devices designed for precise biometry measurements essential in cataract surgery planning. Both instruments operate on the principle of optical biometry, utilizing low-coherence laser beams to scan intraocular structures and attain data on axial length, corneal curvature, and anterior chamber depth.

The key technical difference lies in their underlying technologies for optical coherence. The IOL Master 500 employs partial coherence interferometry (PCI), where interference patterns of reflected light waves are analyzed to extract biometric parameters. In contrast, the IOL Master 700 incorporates swept-source optical coherence tomography (SS-OCT), an advanced imaging technique that utilizes a rapidly turning laser source to acquire high-resolution cross-sectional images of ocular structures.

SS-OCT offers several advantages over PCI, including superior depth resolution, faster acquisition speeds, and reduced susceptibility to signal attenuation in highly scattering tissues. By providing detailed, three-dimensional visualization of ocular anatomy, SS-OCT is believed to enable more accurate and comprehensive biometric measurements, especially in challenging cases such as eyes with dense cataracts, high ametropia, or high irregular astigmatism. Beyond this, the IOL Master 700 is able to perform a total keratometry of the anterior and posterior corneal surfaces. This allows for a precise measurement of corneal curvature and thickness, providing data for accurate intraocular lens (IOL) power calculations and enabling the use of more refined IOL calculation formulas.

## 2. Materials and Methods

### 2.1. Data Collection

The study incorporates a dataset comprising records of 2994 eyes from 1907 patients who underwent cataract surgery. Biometry was performed in-house prior to cataract surgery. Measurements of postoperative refraction were performed by registered ophthalmologists of the ‘South Baden eye network’, which is a network of practices that work together over common IT infrastructure with the University Eye Clinic in Freiburg and provide data such as postoperative refraction and visual acuity. A vote of the ethics committee of the University of Freiburg was obtained before the study.

Key variables collected for each patient are as follows:

The main endpoint of our study was deviation from target refraction as the spherical equivalent. Deviation from target refraction was defined as the absolute difference between manifest and targeted refraction 79 days (mean = 79 days, median = 77 days, min = 1 day/max = 189 days + SD 23 days) after cataract surgery as the spherical equivalent. Spherical equivalent (SE) is a measure of the refractive power of the eye, calculated as the sum of the spherical power (SP) and half of the cylindrical power (CP), expressed mathematically as: SE = SP + (CP/2). When more than one biometric measurement was available, we decided between two cases. When the same target refraction was present, we chose the biometry that was the closest before surgery. If there were different target refractions in the documents, we chose the one that matched our surgery data. 

The covariates in our analysis included the IOL Master version (IOL Master 700 or IOL Master 500), age at the time of measurement, time elapsed between biometry and surgery, gender (male or female), insurance status in terms of private or public health insurance, and the biometrical parameters: anterior chamber depth, axial length, and corneal radius. There were no explicit exclusion criteria regarding the patient cohort. Since we implant the Zeiss CT Asphina 409M IOL almost exclusively (in approximately 98% of cases) at our clinic, we did not include the lens type as a separate factor in our statistical analysis. The Haigis formula was used in nearly all cases, incorporating both axial length and anterior chamber depth. This formula is considered to be accurate over a wide range of ocular anatomies, but as with all formulas, it has limitations in some cases [7,8,9]. The formula was applied with its standard implementation without any customized adjustments. Given the significant proportion of patients with complex ocular conditions at our clinic, tailoring the formula specifically to individual surgeons was not feasible.

### 2.2. Baseline Characteristics

Data from 2017 to 2023 were included due to the good availability of data enabled through a shared software solution. For further information on the characteristics of the patient cohort, a summary of the dataset is presented in Table 1.

**Figure 1:**
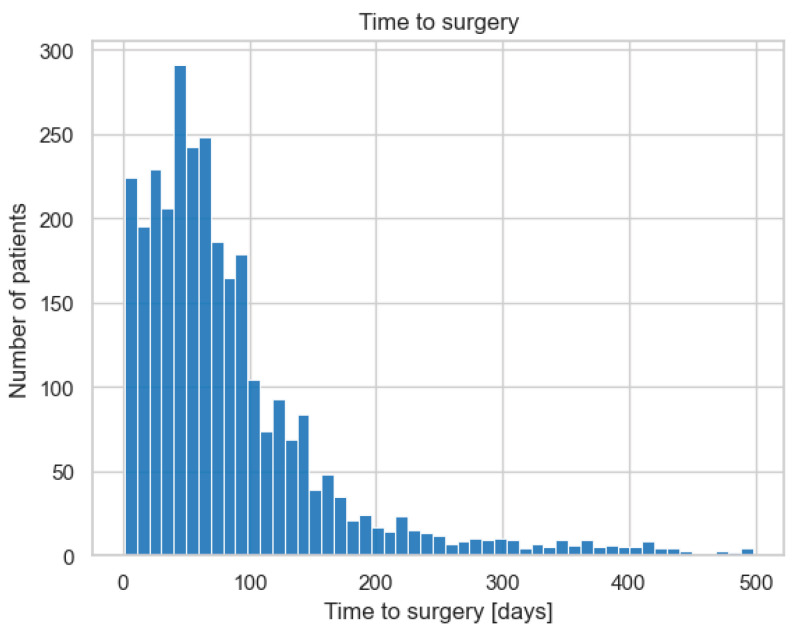
Time between IOL Master device measurement and surgery. Close to 95% of the patients received surgery within 200 days of the measurement.

As a potential confounder, the waiting time until surgery was evaluated and can be seen in Figure 1. The majority of patients were able to receive cataract surgery within 200 days after the indication for surgery was established. 

### 2.3. Statistical Analysis

Multiple regression analysis was employed to assess the relationship between the dependent variable (deviation from target refraction) and the independent variables. The analysis aimed to determine which factors significantly influenced refractive outcomes following cataract surgery. Statistical analysis was performed using the Python programming language with open-source libraries including numpy, pandas, and duckdb. Graphs were created using the libraries matplotlib and seaborn. 

## 3. Results

**Figure 2:**
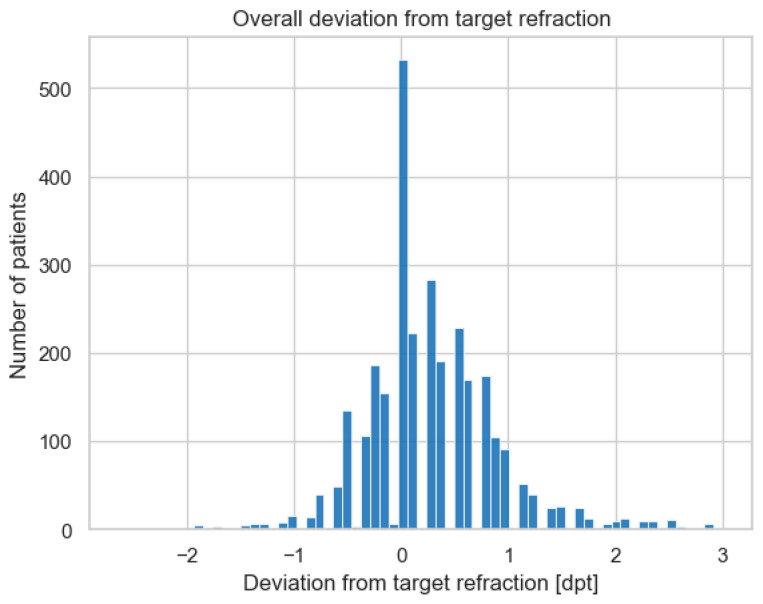
Overall distribution of the deviation from target refraction.

The overall mean deviation from target refraction was −0.3936 dpt, indicating a tendency for slightly myopic outcomes in the study population. The distribution of the deviation from target refraction is illustrated in Figure 2.

**Figure 3:**
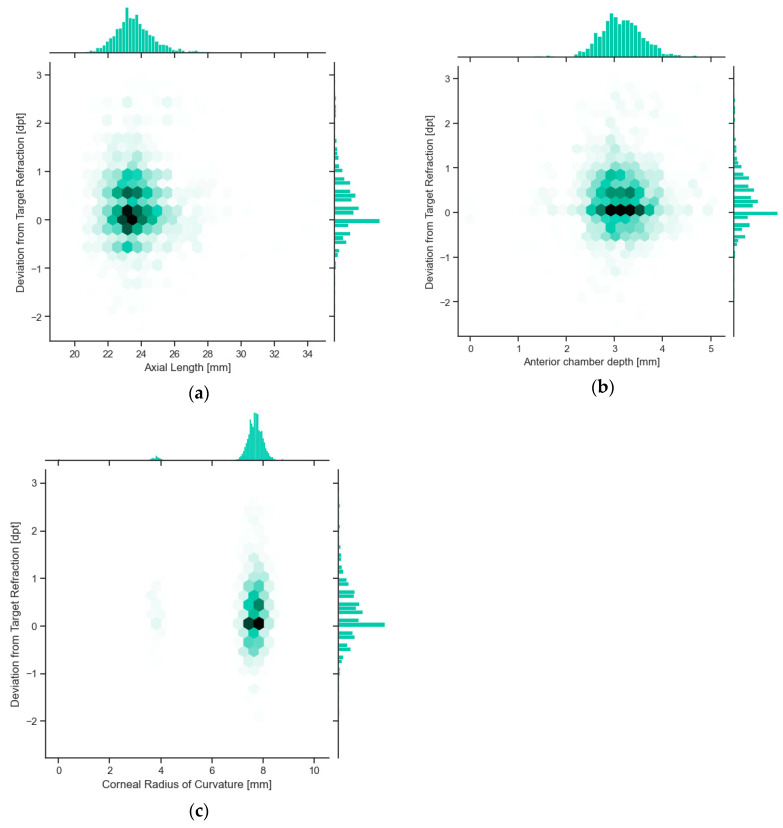
Influence of deviation from target refraction for different biometric parameters. The overall distribution follows the distribution of the three major biometric components. (**a**) Influence of the axial length on the postoperative deviation from target refraction as well as the distribution of the axial length of our patient cohort. (**b**) Influence of the anterior chamber depth on the deviation from target refraction. (**c**) Mean corneal curvature (radius) and the influence on deviation from target refraction.

**Figure 4:**
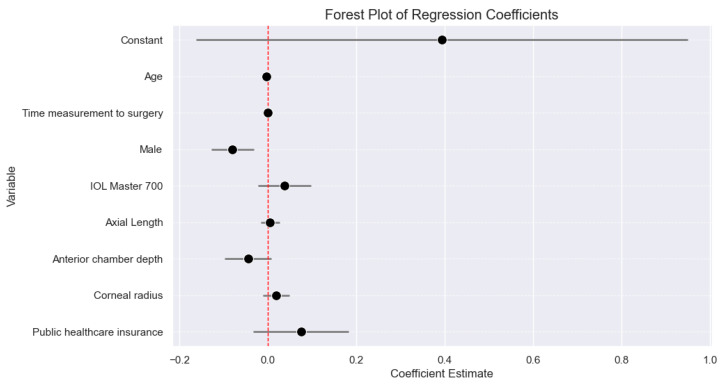
Forest plot for covariants used in our analysis. The target was the difference from target refractions measured as the spherical equivalent.

The multiple linear regression analysis revealed that the constant term, age, and gender were statistically significant predictors of the deviation from target refraction. The constant term had a coefficient of −0.3936 (*p* = 0.164). Age showed a statistically significant association, with a coefficient of −0.0036 (*p* = 0.006), suggesting that increasing age was associated with a slightly more hyperopic outcome. Gender was also a significant predictor, with males having a significantly more hyperopic outcome compared to females, as indicated by a coefficient of −0.079 (*p* = 0.001). The other independent variables, including time elapsed between biometry and surgery, IOL Master version, and insurance status, did not show statistically significant associations with the deviation from target refraction. We also examined the biometric influences on the deviation from target refraction with our multiple linear regression analysis to quantify any potential impacts of the main biometric parameters on deviations from target refraction. The results, detailed in Figure 3 indicate no significant influence of these parameters on refractive outcomes. Specifically, the anterior chamber depth showed a small effect (coefficient: −0.005, *p*-value: 0.623), but this was not statistically significant. Axial length (coefficient: 0.045, *p*-value: 0.088) and corneal radius (coefficient: −0.045, *p*-value: 0.019) did not show a significant effect. The detailed results can be found in Table 2. The resulting forest plot can be found in Figure 4. 

The IOL Master 700 showed a marginal effect in comparison to the IOL Master 500, with patients measured using this equipment tending to have slightly more myopic outcomes, although this finding was not statistically significant (*p* = 0.086). This trend might be attributed to the automatic detection of the fovea by the IOL Master 700.

The time elapsed between biometry and surgery (*p* = 0.489) and insurance status (*p* = 0.172) did not show statistically significant associations with refractive outcomes.

In summary, the multiple regression analysis revealed that age and gender were significant predictors of post-cataract refractive outcomes, while the IOL Master version demonstrated a marginal effect but slightly missed statistical significance. The time between biometry and surgery as well as insurance status did not significantly influence the refractive outcomes in this study population.

## 4. Discussion

Our study aimed to compare the refractive outcomes of cataract surgery patients using the IOL Master 500 and IOL Master 700 for biometry, while also investigating the influence of patient-related factors in a large, real-world cohort. The results suggest that, although both devices are effective for biometry in cataract surgery, subtle differences in their ability to predict refractive outcomes may exist. The IOL Master 700 demonstrated a slight tendency in terms of deviation from target refraction, although this advantage did not reach statistical significance and cannot be considered clinically relevant.

Age and gender were identified as significant predictors of refractive outcomes. Older patients experienced slightly more hyperopic outcomes compared to younger patients, and males had slightly more hyperopic outcomes than females. These findings are consistent with the available literature, which has reported similar effects of age and gender on refractive outcomes following cataract surgery [10]. However, it is important to note that the clinical significance of these differences may be limited, as the effects were observed within a relatively small range.

The consistent offset of 0.3936 observed across all patients, indicating a slight myopic outcome, is most likely to be attributed to the deliberate selection of intraocular lenses (IOLs) aimed at achieving a slightly myopic refractive target. This practice is commonplace in our clinic and is also observed in other eye care facilities. We interpret this consistent deviation as a positive indicator of the quality of the data in our study. The range of inaccuracy of the postoperative refractive outcome is consistent with the literature [11,12,13]. The detection of such a subtle effect underscores the precision and reliability of our measurements.

Our study utilized real-world data, which have inherent limitations, such as the inability to control the method used for measuring postoperative refraction. While objective refraction is assumed to be commonly used due to its availability and efficiency, subjective refraction could also have been employed, potentially leading to differences in refractive outcomes. However, the large sample size of our study helps mitigate the impact of these uncontrolled factors, and the relatively small differences in deviation from target refraction demonstrate the suitability of our approach.

A key differentiating aspect of our work is the lack of exclusion criteria in our data, which we assume includes a wide range of ocular anatomy and cataract types encountered in real-world scenarios, from standard cataracts to more challenging situations. This is a great advantage compared to other studies, which often focus on specific subgroups of patients with different axial lengths and keratometry values.

Furthermore, the IOL Master 700 has been reported to have higher image acquisition rates, especially for patients with dense cataracts. Song et al. found failure rates of 9.7% for IOL Master 500 and 2.4% for IOL Master 700 [1], while Jiang et al. reported even higher values of 17.6% for IOL Master 500 and 6.9% for IOL Master 700 [2]. These findings suggest that the IOL Master 700 may be particularly advantageous for older patients, who are more likely to have dense cataracts.

One limitation of our study is the predominance of a single IOL (Zeiss Asphina 409M) and the low variability of IOL calculation formulas (we mostly used the Haigis formula). While this approach minimizes potential confounding factors and allows for a more robust analysis, it may also limit the generalizability of our findings. Further research is needed to explore these results across different IOL types and calculation formulas. Future studies should also consider including subgroups with extreme ocular biometry to gain a more comprehensive understanding. Given the numerous influential factors involved, we believe that AI-based approaches could be particularly well-suited, especially for handling these special cases.

In conclusion, our study provides valuable insights into the comparative performance of the IOL Master 500 and IOL Master 700 in predicting refractive outcomes following cataract surgery in a large, real-world cohort. Our data, along with evidence from other authors, support the standard procedure of using both devices equivalently as a safe practice for bilateral patients. Age and gender were identified as significant predictors of refractive outcomes, although their clinical significance may be limited. The insights from our investigation will help optimize biometry for cataract surgery for safe patient care.

## Figures and Tables

**Table 1:** Baseline characteristics of our patient cohort.

Property	Value
Included number of patients	2994 eyes, 1907 patients
Sex	1300 (M)/1694 (F)
Age	74.4 years
Insurance status	2864 (95.7%) regular/130 (4.3%) private
Time between measurement and surgery	Mean: 85 days, min: 1 day, max: 498 days, SD: 79 days

**Table 2:** Detailed results from our multiple linear regression analysis.

Variable	Coefficient	Standard Error	t	*p* > |t|	[0.025	0.975]
Constant	0.3936	0.283	1.391	0.164	−0.161	0.948
Age	−0.0036	0.001	−2.769	0.006	−0.006	0.001
Timespan	9.628 × 10^−5^	0	−0.692	0.489	0.000	0.000
Sex male	−0.0799	0.024	−3.341	−0.127	−0.127	−0.033
IOL Master type	0.0375	0.030	1.268	0.205	−0.021	0.096
Axial length	0.0050	0.010	0.492	0.623	−0.015	0.025
Anterior chamber depth	−0.0448	0.026	−1.709	0.088	−0.096	0.007
Mean corneal radius	0.0191	0.014	1.327	0.185	−0-009	0.047
Insurance regular	0.0522	0.056	1.365	0.172	−0.058	0.162

## Data Availability

Due to potential data privacy considerations, the dataset used in this study is not publicly accessible. However, upon request, we are prepared to furnish additional data as needed.
